# Risk Factors and Stroke Subtyping in Young Adults: A Study From a Tertiary Care Hospital in South India

**DOI:** 10.7759/cureus.63640

**Published:** 2024-07-02

**Authors:** Sonia Shivde, Sagar Badachi, Saikanth Deepalam, Raghunandan Nadig, Akshata Huddar, Thomas Mathew, GRK Sarma, Sharath Kumar GG, Swathi S Sanjee, Sreerag Kapparath

**Affiliations:** 1 Neurology, St. John's National Academy of Health Sciences, Bengaluru, IND; 2 Intervention Neuroradiology, St. John's National Academy of Health Sciences, Bengaluru, IND; 3 Neurology, St. John's National Acedemy of Health Sciences, Bengaluru, IND; 4 Radiology, St. John's National Academy of Health Sciences, Bengaluru, IND; 5 Neurology, St. John's Academy of Health Sciences, Bengaluru, IND

**Keywords:** mrs, nihss, icad, toast classification, young adults, stroke

## Abstract

Objectives: This study aimed to determine the risk factors and stroke subtypes for young ischemic stroke patients and their outcomes at the time of discharge.

Methods: This is a retrospective cross-sectional study of ischemic stroke patients (n = 264) between the age groups of 18 and 45. The study population was divided into two broad age groups: 18 to 35 years and 36 to 45 years; and compared based on demographics, risk factors, the Trial of Org 10172 in Acute Stroke Treatment (TOAST) classification, and outcomes. The outcomes were compared based on the National Institutes of Health Stroke Scale (NIHSS) and Modified Rankin Scale (MRS) systems at the time of admission and discharge.

Results: The mean age of patients was 37.84±6.19 years. The male-to-female ratio was 2.5:1. The most common vascular risk factors identified were diabetes (29.16%), hypertension (49.62%), dyslipidaemia (DLP, 44.4%), and smoking (10.9%). The most common TOAST subtype was large vessel disease (38.63%), followed by the undetermined category (35.6%). The elderly group showed a high proportion of strokes secondary to small vessel disease (14.13%; p = 0.03), while cardioembolic strokes were common in the female subgroup (p = 0.05). The majority of strokes were in the anterior circulation (66.6%) as compared to the posterior (25.75%), and nearly 50% of the patients had intracranial disease. Overall, there was a favourable MRS outcome at discharge.

Conclusion: Conventional vascular risk factors are equally prevalent, even among young stroke patients. The benchmark for young stroke age is showing a downward shift as more stroke patients above the age of 35 are showing similar risk factor trends as those of their older counterparts. The majority of stroke burden still falls under the undermined category, which requires aggressive risk factor identification and management.

## Introduction

Stroke is one of the major causes of premature death and disability in developing nations like India [1]. The burden of stroke, especially when it occurs in an economically productive age group, can have a dramatic impact. The incidence of stroke in young people has risen in the last few years. A study by Kissela et al. observed trends of increased incidence of stroke at younger ages [2]. Though research in the last two decades on the incidence and etiological factors for young stroke has contributed to our knowledge, there is a lot of heterogeneity in various studies, as few are population-based, few are hospital-based, some in various time frames, some have racial and ethnic differences, etc., because of which the results are conflicting. Also, the majority of these studies are from the Western world, and we still lack enough Indian data from various regions for comparison. As per the systematic review by Smajlović, stroke in young adults comprises 10% to 15% of all stroke patients [3].

The lower age limit for conventional modifiable risk factors has shifted significantly to encompass a much younger population. This poses them with the risk of adverse ischemic events, both cardiac and cerebrovascular. It is well known that conventional risk factors have a high prevalence in young strokes, in addition to their uncommon aetiologies, as per various studies across the world. Primary prevention of these risk factors can significantly help to unload the stroke burden. Timely risk factor identification, control of hypertension, diabetes, and dyslipidemia, a healthy lifestyle, moderate aerobic physical activity and exercise, smoking abstinence, and limited consumption of alcohol can help reduce the morbidity and mortality related to stroke.

Though prevention is undoubtedly the primary treatment strategy in stroke management, the importance of secondary prevention cannot be denied. Stroke is a major area of neurology where timely interventions can have fruitful outcomes. Due to the high prevalence of vascular risk factors, large artery atherosclerosis is one of the major causes of stroke in young people. Knowing the prevalence of various aetiologies is thus crucial in the planning and implementation of treatment strategies. However, the aetiology of stroke in young people is diverse and differs in various geographical regions. The Trial of Org 10172 in Acute Stroke Treatment (TOAST) model of classification for ischemic strokes is used in the majority of the studies. Our study is a hospital-based study from a tertiary healthcare centre in Bengaluru, South India, to determine the risk factors and aetiology of ischemic strokes in young patients using TOAST classification. This will shed some light on trends in the aetiology of stroke in the younger population in this geographical region, which will help in the revision of treatment protocols and stroke prevention programmes.

## Materials and methods

Aims and objectives

This study aimed to investigate the risk factors associated with strokes among young people, as well as the correlation between these risk factors and stroke subtypes based on the TOAST classification of young strokes.

Study design and sample recruitment

This is a retrospective cohort study. It was conducted at the neurology department of St. John's National Academy of Health Sciences, Bengaluru, India, a tertiary care hospital in South India, after approval from the institutional ethics committee (IEC study reference no. 223/2018). We reviewed the inpatient charts and discharge summaries of patients admitted with ischemic stroke from 2016 to 2022. Out of 1,500 patients who were admitted and diagnosed with ischemic stroke during this period, we selected patients aged between 18 and 45 years. Previous studies done in young stroke patients, including Indian data, considered an age cutoff of either 45 or 49 years [4,5]. Therefore, this age group was included for better comparison. Written informed consent was obtained from all the study participants.

Inclusion criteria

Patients admitted with ischemic strokes within the age group of 18 to 45 years with or without a history of ischemic strokes were included in the study.

Exclusion criteria

Patients with cortical venous thrombosis, ischemic strokes secondary to direct head trauma, and intracranial bleeds, including subarachnoid haemorrhage, were excluded from the study.

Methodology

Basic laboratory investigations such as complete blood counts, serum electrolytes, random blood sugar, glycated haemoglobin (HbA1c), liver and renal function tests, HIV, venous disease research laboratory test (VDRL), prothrombin time (PT) test/international normalised ratio (INR), vitamin B12 levels, etc. were performed for all stroke patients as a routine part of care. A baseline ECG was done for all patients. The majority of patients except a few underwent echocardiogram testing, with some patients undergoing transthoracic or bubble echo if recommended by their treating neurologist. The Holter examination was performed whenever considered appropriate. Serum homocysteine, anti-nuclear antibody, and antiphospholipid antibody (ALPA) were done in most of the young stroke patients as part of the protocol. Few patients underwent detailed prothrombotic workups. An MRI of the brain was performed in all patients except five patients who underwent CT imaging. All patients underwent vascular imaging in the form of a CT or MR angiogram, except 12 patients. All scans were reviewed carefully by treating neurologists, and additional input was sought from the neuroradiologist team, especially in the case of vascular imaging.

All the medical records were reviewed by the neurologist for history, demographic profile, personal habits, history of transient ischemic attack (TIA) or stroke, clinical examination, and treatment received. The data were entered into the predesigned Microsoft Excel sheet (Microsoft Corp., Redmond, WA). We broadly divided the patients into two age groups, 18 to 35 years and 36 to 45 years, to compare the trend of risk factors in both groups. The TOAST classification system for ischemic stroke was used to compare the stroke aetiology between these two groups. The National Institutes of Health Stroke Scale (NIHSS) score and Modified Rankin Scale (MRS) were compared at the time of admission and discharge.

Definitions Used

Ischemic stroke is an episode of focal neurological deficits with acute onset and lasting >24 hours (or lasting <24 hours with imaging evidence of stroke corresponding with current symptoms); TIA was defined similarly but with symptoms lasting <24 hours and without corresponding imaging evidence of an ischemic lesion.

Statistical analysis

Data were expressed as mean and standard deviation in a normal distribution and median with 25^th^ and 75^th^ percentiles for a non-normal distribution. The data were expressed in numbers with percentages for the categorical variables. Continuous variables were compared using the t-test, and the chi-square or Fischer's exact test was applied for categorical variables. A p-value less than 0.05 was considered statistically significant. All the analysis was performed using IBM SPSS Statistics software for Windows, version 25.0 (IBM Corp., Armonk, NY).

## Results

Two hundred and sixty-four patients belonging to the age group of 18 to 45 years were included in the study. The mean age of the patients was 37.84±6.19 years. The most common TOAST subtype in this study was large vessel disease (38.63%), followed by the undetermined category (35.6%). The most common risk factors identified and stroke subtyping are depicted in Table 1.

**Table 1:** Risk factors, stroke subtyping, and stroke location (n=264) TOAST: Trial of Org 10172 in Acute Stroke Treatment; ODC: other determined causes; TC: total cholesterol; HDL: high-density lipoprotein; TIA: transient ischemic attack

	Frequency (n = 264)	Percentage
Age groups (in years)		
18 – 35	80	30.3 %
36 – 45	184	69.69 %
Gender		
Male	188	71.21%
Female	76	28.78%
Stroke location		
Anterior	176	66.66%
Posterior	68	25.75%
Both territories	20	7.57%
TOAST subtypes		
Large vessel	102	38.63%
Small vessel	30	11.36%
Cardioembolic	27	10.22%
ODC	11	4.16%
Undetermined	94	35.6%
Risk factors		
Diabetes	77	29.16%
Hypertension	131	49.62%
TC (>200mg/dL)	62	26.26%
(TC/HDL ratio >5.0)	105	44.49%
Ischemic heart disease	13	4.92%
Rheumatic heart disease	16	6.06%
Cardiomyopathy	4	1.51%
Cardiac conduction abnormality	3	1.13%
Smoking	29	10.98%
Alcohol	42	15.9%
History of TIA/stroke	25	9.49%
Hyperhomocysteinemia	39	17.18%
B12 deficiency	85	32.94%

Stroke of undetermined origin (41.2%) was the most common subtype among the younger age group (18 to 35 years), while strokes associated with large vessel disease (38.04%) predominated the latter (36 to 45 years). Strokes of other determined causes (ODC) were the least common among both age groups (Table 2). The comparison between the two major age groups for conventional risk factors did not show a significant statistical difference (Table 3). Males had a significantly higher proportion of large vessel disease as compared to females. Among females, a stroke of undetermined aetiology was the most common subtype observed. Females had a higher number of cardioembolic strokes as compared to males (Table 2). In the ODC category, four patients had arterial dissections, three patients were APLA-positive, and the rest had protein S deficiency, systemic lupus erythematosus (SLE) vasculitis, Moya Moya disease, and an MTHFR gene homozygous mutation.

**Table 2:** The TOAST classification stratified based on age and gender (n=264) TOAST: Trial of Org 10172 in Acute Stroke Treatment; ODC: other determined causes

TOAST subtype	18-35 (n=80)	36-45 (n=184)	Total (n=264)	P-value
Cardioembolic	8(10%)	19(10.32%)	27(10.22%)	0.935
Large vessel	32(40%)	70(38.04%)	102(38.63%)	0.764
Small vessel	4(5%)	26(14.13%)	30(11.36%)	0.031
ODC	3(3.75%)	8(4.34%)	11(4.16%)	0.823
Undetermined	33(41.25%)	61(33.15%)	94(35.6%)	0.206
TOAST subtype	Female (n=76)	Male (n=188)	Total (n=264)	P-value
Cardioembolic	12(15.78%)	15(7.97%)	27(10.22%)	0.050
Large vessel	19(25%)	83(44.14%)	102(38.63%)	0.0038
Small vessel	11(14.47%)	19(10.10%)	30(11.36%)	0.311
ODC	9(11.84%)	2(1.06%)	11(4.16%)	0.745
Undetermined	25(32.89%)	69(36.70%)	94(35.6%)	0.558
Cardioembolic	12(15.78%)	15(7.97%)	27(10.22%)	0.050

**Table 3:** Comparison between two major age groups for conventional risk factors (n = 264) DLP: dyslipidaemia; TC: total cholesterol; HDL: high-density lipoprotein; IHD: ischemic heart disease; RHD: rheumatic heart disease

Risk factors	18-35(n=80)	36-45(n=184)	P value
Hypertension	24(30%)	107(58.1%)	2.61
Diabetes	7(8.75%)	70(38.0%)	1.49
DLP (TC/HDL ratio>5)	29(36.2%)	80(43.4%)	0.27
IHD	6(7.5%)	7(3.80%)	0.20
RHD	3(3.75%)	10(5.43%)	0.56

Patients underwent vascular imaging in the form of a CT or MR angiogram. Selected patients underwent a digital subtraction angiogram (DSA). Strokes in the anterior circulation were more common (176, 66.6%) than those in the posterior circulation (68, 25.75%). Both territories were involved in 20 (7.57%) cases. Strokes due to large vessel atherosclerosis predominated in the anterior circulation as compared to the posterior circulation (p = 0.04). The most common TOAST subtype in posterior circulation was undetermined aetiology (p = 0.02) (Table 4).

**Table 4:** TOAST subtyping according to stroke locations TOAST: Trial of Org 10172 in Acute Stroke Treatment; ODC: other determined causes

TOAST subtype	Anterior (n=176)	Posterior (n=68)	Total (n=244)	P value
Cardioembolic	18(10.22%)	3(4.41%)	21(8.60%)	0.042
Large vessel	79(44.88%)	21(30.88%)	100(40.98%)	0.046
Small vessel	22(12.5%)	8(11.76%)	30(12.29%)	0.875
Undetermined (Incomplete)	55(31.25%)	32(47.05%)	87(35.65%)	0.020
ODC	2(1.13%)	4(5.88%)	6(2.45%)	

Seventy-nine patients had significant atherosclerotic disease in the anterior circulation. Out of these, extracranial (EC) vessels were involved in 28 cases, intracranial (IC) vessels were involved in 43 cases, and both IC and EC involvement was seen in eight cases. Two patients in the EC group had critical stenosis of both internal carotid arteries (ICAs). Twenty-one patients had large artery atherosclerosis (LAA) in the posterior circulation, out of which 11 had involvement of EC vertebral artery disease and 10 had IC vertebrobasilar disease. Four patients in the ODC category had arterial dissections, of which one was in the ICA and three in the vertebral arteries. 

A 2D echo test was performed in all cases except 15 patients and was normal in 210 (84.3%) patients. Additionally, a transoesophageal echocardiogram (TEE) was performed in three cases and a bubble echo in two cases. The most common abnormality detected was rheumatic heart disease (RHD), with valvular defects in 16 (6.06%), ischemic heart disease (IHD) in 13 (4.92%), dilated cardiomyopathy (DCMP) in four (1.51%), atrial fibrillation (AF, both valvular and non-valvular) in three (1.13%), and congenital heart disease in two (0.75%) cases. Two patients with patent foramen ovale (PFO) were confirmed with TEE. Few patients underwent Holter testing.

Nineteen (7.1%) patients underwent thrombolysis, of which alteplase was used in 11 cases and tenecteplase in four cases. Two patients underwent mechanical thrombectomy. Two patients underwent ICA stent placement in the same setting. It was observed that more than 70% of patients with a history of stroke or TIA were incompatible with antiplatelet or anticoagulation therapy. Ten patients who were known cases of RHD had subtherapeutic INR at the time of presentation. Sixty percent of patients were discharged on dual antiplatelet therapy (DAPT), while 17% received single antiplatelets (SAPT). 22% of patients received a single antiplatelet and oral anticoagulation combination. Patients who underwent ICA stenting were initiated on ticagrelor.

The NIHSS and MRS scores were compared, respectively, at the time of admission and discharge. An NIHSS score of five or less at the time of admission and discharge was 53.4% and 79.54%, respectively. A MRS score of 0 to two at the time of admission was observed in 49.2% of cases, which increased to 72.7% at the time of discharge. The in-hospital mortality rate was 2.6% (Table 5).

**Table 5:** Comparison between the NIHSS and mRS scores at admission and discharge NIHSS: National Institutes of Health Stroke Scale; MRS: Modified Rankin Scale

NIHSS (admission)	18-35(n=80)	36-45(n=184)	Total(n=264)	Percentage (%)
<5	41(51.2%)	100(54.34%)	141	53.4
5-15	38(47.5%)	80(43.4%)	118	44.69
>15	1(1.25%)	4(2.17%)	5	1.89
NIHSS (discharge)				
<5	58(72.5%)	152(82.60%)	210	79.54
5-15	21(26.25%)	30(16.30%)	51	19.31
>15	1(1.25%)	2(1.08%)	3	1.13
MRS (admission)				
0-2	37(46.25%)	93(50.54%)	130	49.24
3-4	38(47.5%)	85(46.19%)	123	46.59
5-6	5(6.25%)	6(3.26%)	11	4.16
MRS (discharge)				
0-2	53(66.25%)	139(75.54%)	192	72.72
3-4	24(30%)	41(22.28%)	65	24.62
5-6	3(3.75%)	4(2.17%)	7	2.65

## Discussion

The WHO estimates for the last two decades indicate that stroke-related mortality is significantly higher in low- and middle-income countries (LMICs) [6]. Strokes among the young comprise about 10% to 15% of stroke cases, and a population-based study has shown that the stroke incidence rate in the age group 20 to 50 shows a significant increase in the last two decades as compared to the previous period [2,3]. Identifying the aetiology and risk factors of young strokes is thus crucial for the accurate classification of stroke, which can help in proper management and future prevention.

The mean age in our study was 37.8 years, which is similar to the retrospective study from North India by Dash et al. [7]. We found male predominance in our study, which parallels the findings in most other Indian studies [8,9]. This finding is in contrast with the Western world, where the ratio is nearly equal or shows a slight male preponderance [10,11]. Apart from the commonly known cultural factors due to which males are more likely to seek healthcare at tertiary care centres as compared to females, we also noted that conventional risk factors were more common in males, like smoking, alcohol, hypertension, etc. We found hypertension to be the most commonly identifiable risk factor, followed by diabetes. These results are similar to the population-based prospective cohort study done at Ludhiana, where the incidence of hypertension and diabetes among young strokes was 72% and 23%, respectively [5]. Evidence from Western literature suggests that the association of traditional vascular risk factors with young strokes is stronger [12]. In the study conducted in the Baltimore-Washington area, the odds ratio (OR) for diabetes mellitus (DM) in White males was as high as 22.9 [13]. This study concluded that hypertension, DM, and cigarette smoking were important risk factors for biracial young patients. Dyslipidaemia (DLP) was another important risk factor observed in our study, with 44.4% of patients showing a total cholesterol (TC)/high-density lipoprotein (HDL) ratio >5.0 and 26% showing TC >200 mg/dl. These findings were similar to those of the other Indian studies [7]. In a Ludhiana population-based study, DLP was observed in 15% of young strokes [5]. Various studies from European countries reported DLP rates ranging from 26.4% to 52.7% [10, 11, 14]. Our study reports a lower frequency of smoking as compared to the data from the Western world [11,15]. The incidence of smoking ranged from 9% to 49% [7,16].

The current study reported LAA as the most common subtype, followed by strokes of undetermined aetiology. These results are similar to those of another study from South India [17]. A study from North India reported undetermined aetiology as the most commonly observed subtype [7]. Large artery atherosclerosis dominated among males in our study (p = 0.0038), and this could be attributed to the higher percentage of conventional risk factors in males. Females had an undetermined aetiology as the most common stroke subtype. The reason for this is the incomplete evaluation in the majority of cases. As per the previous literature, cardioembolic strokes are more commonly reported among young females, and the most common aetiology is RHD. A case-control study conducted in South India by Lipska et al. showed cardioembolic strokes as the most common TOAST subtype [9]. The majority of the Indian studies, including the current study, had RHD as the most common aetiology among cardioembolic strokes [7,17,18]. Despite a significant decline in RHD prevalence over the last century, it continues to be a burden requiring adequate clinical and public health responses [19]. A TEE is still not widely available in our country, and in the available settings, its use is still limited. Only three patients in our study underwent TEE. Real-time TEE in patients with normal TEE can help in reclassifying patients in the undermined category by increasing the yield of the pick rate of the left ventricle (LV), left atrial (LA) clots, and PFO. Western literature reports PFO and atrial septal aneurysm (ASA) as the most common aetiologies behind cardioembolic strokes [14,20].

Anterior circulation (AC) strokes were more common than posterior circulation (PC) strokes. Though there is limited evidence, it is observed that conventional risk factors exert varying magnitudes of impact on ischemic stroke among different arterial territories. Risk factors such as DM and hypercholesterolemia dominate the PC territory, while hypertension does not have any specific arterial predilection [21]. We found that LAA and cardioembolic strokes were more frequent in the AC. These results are aligned with a study conducted by Di Carlo et al., which showed a higher percentage of cardioembolic subtypes and AF in AC strokes [22]. Our study observed that the most common stroke subtype in PC territory had an undetermined aetiology. This may be due to multiple reasons. As strokes due to more than two aetiologies are classified under an undetermined category, it is possible that many such cases might be included in this group. Apart from this, rare cases like mitochondrial cytopathies and Fabry disease, which have a predilection for PC, might have been missed due to an incomplete evaluation [23]. In contrast to the above findings, a US study did not observe any significant differences in demographics, risk factors, or stroke subtypes between the two groups [24]. Though, one of the important causes of young strokes, our study underreported arterial dissections and APLA syndromes. As compared to our study, a higher rate of APLA positivity was observed in one South East Asian young stroke cohort [25]. As a routine part of care in our institute, we consider APLA positivity only after double confirmation with two tests done 12 weeks apart due to the high rate of false positivity. As only three patients were double-confirmed positive, the possibility of underreporting may exist.

Our patients showed overall good functional outcomes at the time of discharge. Nearly 72% of patients had MRS scores of two or below at the time of discharge. These findings are similar to the study conducted by Nedeltchev et al., which considered MRS of 0 to one as a favourable outcome in the young stroke population [26]. While MRS of two (the ability to look after one’s affairs without assistance) at the time of discharge may sound like a favourable outcome in the case of the elderly, that may not be the case for functional young adults. Another study found that nearly 94% of patients had an MRS of 0 to two on subsequent follow-ups [27]. Conversely, few studies and case series reported unfavourable outcomes in young strokes [28,29]. High NIHSS scores at the time of admission and DM were independent risk factors for poor outcomes in these patients [26].

A secondary stroke prevention strategy to prevent further recurrence of strokes in the younger population comes with its own set of challenges. A study by Homma et al. observed a 9.4% five-year recurrence risk in young patients [30]. The risk factors associated with the recurrence are traditional vascular risk factors like hypertension, DM, history of TIA, LAA as the primary aetiology, and cardiac failure [31]. Though traditional risk factors seem to play an important role, many cryptogenic strokes may have varied aetiologies, like PFO, which may be missed in routine evaluation [30]. The stroke aetiology strongly influences the decision to start antiplatelets or add anticoagulation. Sixty percent of our patients were discharged on DAPT. The three main indications for starting DAPT are 1) high risk of TIA; 2) minor ischemic stroke (NIHSS<4); and 3) stroke resulting from symptomatic intracranial atherosclerotic disease (ICAD)[32]. Our study showed that nearly 50% of patients in the LAA group had significant ICAD in both the anterior and posterior circulations. The DAPT was given for a minimum duration of 90 days due to its high-risk profile and considering the results of the Stenting and Aggressive Medical Management for Preventing Recurrent Stroke in Intracranial Stenosis (SAMMPRIS) trial [33,34]. As young strokes are a heterogeneous group and the diagnostic workup is broad, more studies are required in the future to consolidate the secondary preventive strategies.

The strength of this study is the large, consecutive sample size gathered over the last half of a decade. As our hospital is a comprehensive stroke care centre, patients were analysed and treated as per the latest stroke guidelines, including comprehensive magnetic resonance imaging with stroke protocol. Due to the in-house availability of catheterization (cath) lab and thrombolysis services, timely interventions and outcome assessment were feasible. The limitations of our study include the retrospective cohort, which was followed up over a longer period of time. Hence, the work-up may vary during different time frames. Also, many patients did not undergo a thorough workup for the identification of stroke aetiology. This study is carried out at a tertiary care institute; hence, there is a possibility that we may get more referral cases, and hence the data may not be extrapolated to the general population.

## Conclusions

Traditional risk factors commonly associated with strokes in the elderly population play a pivotal role in the aetiology of young strokes as well. Despite this well-known fact, the cause of stroke in many younger patients remains obscure. The current classification system applied routinely for elderly strokes (age >50 years) fails to identify the cause in many young patients, and the majority of these strokes are identified as cryptogenic. This highlights the need for a newer classification system for strokes in young people. Many potential risk factors can be identified in such cases by expanding the stroke subgrouping and applying an extended battery of investigations.
